# Wherefrom and Whither PD? Recent Developments and Future Possibilities in *DSM-5* and *ICD-11* Personality Disorder Diagnosis

**DOI:** 10.1007/s11920-025-01602-y

**Published:** 2025-03-20

**Authors:** Lee Anna Clark

**Affiliations:** Department of Psychology, University of Notre Dame, 390 Corbett Family Hall, IN 46556 Notre Dame, France

**Keywords:** Personality disorders, *DSM-5*, *ICD-11*, Assessment and diagnosis, Dimensional models

## Abstract

**Purpose of Review:**

Provide an overview of the Alternative *DSM-5* model of Personality Disorder (AMPD) and *ICD-11*’s PD diagnostic model; review the models’ assessment measures and construct validity; describe the models’ current and ongoing status.

**Recent Findings:**

The models have many content similarities but differ significantly in that maladaptive-range traits are an AMPD requirement, but optional specifiers in ICD-11. An extensive literature using the Personality Inventory for *DSM-5* (PID-5) has yielded comprehensive reviews of its clinical utility and construct validity. Structural meta-analyses found three core facets for each of the five domains, and correlations with non-FFM measures identified 13 traits with maladaptive content not assessed by the PID-5. Joint analyses of AMPD personality-functioning measures find a clear general factor, but have raised concerns about discriminant validity among measures and with Criterion B. For both criteria, the multimethod assessment literature is sparce. Regarding ICD-11, few measures assess the required personality functioning, but one has shown promising construct validity. Multiple measures assess the optional trait specifiers, the most noteworthy of which was developed by an international group, exists in 12 languages, and assesses all six domains of the *DSM-5* and *ICD-11*.

**Conclusion:**

The status of the ongoing revision process for the AMPD is described. It seems likely—but far from guaranteed—to result in a dimensional model in the main *DSM-5* section. The next step for the *ICD-11* is development of a version with Research Diagnostic Criteria, but the timeline is unknown.

## Introduction

Both the *DSM-5* [1] and *ICD-11* [2] include, for the first time a dimensional diagnostic models for personality disorder (PD). In the former, the model is included in Section III (Emerging Models and Methods) as an alternative (typically abbreviated AMPD) to the traditional categorical model, whereas in *ICD-11*, it replaces the previous categorical model. The AMPD has now accumulated a decade’s worth of research that has generally supported its theoretical basis, empirical validity, and clinical utility, so is poised to replace the traditional model in the main *DSM-5* Section II. In contrast, the *ICD-11* PD model is quite new, but its research and clinical base will no doubt grow over the coming years, as the *ICD-11* is used globally. In this article, I provide an overview of each model and discuss their similarities and differences. I also describe existing measures developed to assess them, the next step in developing the *ICD-11* model, and the ongoing process by which a version of the AMPD may replace the current model in *DSM-5* Section II.

### Diagnostic and Statistical Manual of Mental Disorders, 5^th^ Edition (DSM-5) [1]

The *DSM-5* includes two distinct diagnostic models for personality disorder (PD). The *DSM-5*’s main body (Section II) retains a traditional, categorical model of 10 putatively distinct PDs, whereas its Section III (Emerging Models and Methods) introduces an alternative, dimensional model for PD (AMPD), consisting of two main criteria—personality functioning and personality traits—both of which range from the adaptive to the maladaptive range. A PD diagnosis is made only if there is moderate or greater impairment in personality functioning and one or more personality traits in the pathological range. Five additional criteria (C through G) further define the two main criteria (e.g., “the impairments in personality functioning and the individual’s personality trait expression are relatively inflexible and pervasive across a broad range of personal and social situations,” [1, p. 761]).

In the AMPD, personality functioning (Criterion A) is conceptualized as having two main components—self functioning and interpersonal functioning; self functioning involves identity and self-direction, whereas interpersonal impairment involves intimacy and empathy. Personality traits (Criterion B) consists of 25 continuous dimensions called *facets*, which are organized into five broad dimensions termed *domains*: Negative Affectivity (NA), Detachment (DET), Antagonism (ANT), Disinhibition (DIS), and Psychoticism (PSY). Each domain is indexed by three specific facets [3]; the other 10 facets can be used for further characterization of individuals’ personality traits. For example, ANT’s core facets are Manipulativeness, Deceitfulness, and Grandiosity, whereas Attention Seeking, Callousness, and Hostility may additionally characterize some individuals who manifest pathological-range ANT.

In addition, the AMPD provides diagnostic criteria for six specific PDs—antisocial, avoidant, borderline, narcissistic, obsessive–compulsive, and schizotypal PD. For the two main criteria of these PDs, a particular constellation of personality impairments and maladaptive traits that indicate that specific PD are described. For example, Identity in Antisocial PD’s Criterion A is “Egocentrism; self-esteem derived from personal gain, power, or pleasure” [1], p. 764, whereas for Avoidant PD it is “Low self-esteem associated with self-appraisal as socially inept, personally unappealing, or inferior; excessive feelings of shame” [1], p. 765.

Similarly, Obsessive–Compulsive PD’s Criterion B requires a pathological level of three of the following four trait facets: Rigid perfectionism, Perseveration Intimacy Avoidance, and Restricted Affectivity, whereas that for Schizotypal PD requires four or more of six trait facets: Cognitive and Perceptual Dysregulation, Unusual beliefs and Experiences, Eccentric Perceptions (all three of which are facets of the Psychoticism domain), Restricted Affectivity, Withdrawal, and Suspiciousness.

Moreover, some facets are modified to reflect a specific PD more precisely. For example, the description of trait facet Withdrawal for Avoidant PD, omits the phrase “preference for being along to being with others,” while retaining the other descriptors: “reticence in social situations; avoidance of social contacts and activity; [and] lack of initiation of social contact” [1], p. 766. Lastly, if an individual has “one or even several prominent traits that may have clinical relevance in addition to those required for a specific PD, the option exists for these to be noted as specifiers” [1] p. 771.

Finally, an AMPD diagnosis of Personality Disorder–Trait Specified (PD-TS) is made if the AMPD’s General Criteria are met, but “the personality functioning or trait pattern is substantially different from that of any of the six specific PDs” [1] p. 771. This diagnosis is used when the clinical presentation is a mixture of, or atypical for, those of the specific PDs, including clinical presentations that would be diagnosed with Section II Paranoid, Schizoid, Histrionic, or Dependent PD, or those of previous *DSM*s (e.g., Passive-aggressive PD). Notably, given the high comorbidity among the AMPD’s specific PDs, it has been argued that they are an unnecessary complication of the model, and that PD-TS alone is sufficient [4]. Moreover, Clark and colleagues [5] presented “strong evidence that the AMPD yields the same overall prevalence of PD as the current model and, further, identifies largely the same overall population. It also … provides more complete, individualized characterizations of persons with PD” [5] p. 2210. Taken together, these findings provide compelling arguments for simplifying the AMPD by eliminating the specific PDs.

### International Classifications of Diseases, 11^th^ Edition (ICD-11) [2]

The *ICD-10* [6] model for PD was a categorical model like that of *DSM-5*, Section II, with most of the same specific PDs (although the labels differed in several cases, e.g., Anankastic PD rather than Obsessive–Compulsive PD). However, in the *ICD-11* [5], the PD model became dimensional, with many similarities to the AMPD including (1) impairment in personality functioning—“disturbance” in *ICD-11*—is an essential (required) feature, as are (2) maladaptive patterns in cognition, emotional experience and expression, and behavior (i.e., traits, as long as they meet other criteria for persistence and pervasiveness, etc.); (3) each has three severity levels of impairment/disturbance above the diagnostic threshold; (4) the AMPD’s general criteria C through G and five of *ICD-11* PD’s essential features are virtually paraphrases of each other; and (5) the impairment/disturbance is associated with (“leads to” in the AMPD) difficulties in social, occupational, and other important areas of life.

However, there also are notable differences. Most notably, (1) *ICD-11*’s essential features tend to be more descriptive than the more prescriptive AMPD criteria (i.e., a non-exhaustive list of characteristics in the *ICD-11* model vs. e.g., “*manifested* by characteristic difficulties in *two or more of the following four areas*” in the AMPD); (2) five maladaptive-range traits are provided as optional specifiers rather than included among the essential (required) features; (3) only four of each model’s five traits are shared—NA, DET, ANT (call Dyssocial in *ICD-11*), and DIS—PSY is not among the *ICD-11* traits because Schizotypal Disorder is placed in the Schizophrenia and Related Psychoses chapter in *ICD-11* and Anankastia, which encompasses traits relevant to obsessive–compulsive PD such as rigid perfectionism and behavioral and emotional constraint; and (4) the *ICD-11* model also provides an optional Borderline Pattern specifier that may be applied to individuals whose pattern of personality disturbance is characterized by a pervasive pattern of instability of interpersonal relationships, self- image, and affects, and marked impulsivity as indicated by many of the criteria for *DSM-5*, Section II Borderline personality disorder. See Table 1 for a detailed side-by-side comparison of the two models.

**Table 1:** Comparison of *DSM-5* and *ICD-11* models of personality disorder

*DSM-5*	*ICD-11*
**Conceptualization and Definitions**	
**Criterion A**	**Essential Feature**
Impairment in personality (self- and interpersonal) functioning	Problems in functioning of aspects of the self …and/or interpersonal dysfunction
Self-functioning components: identity and self-direction	
Interpersonal-functioning components: empathy and intimacy 12 subcomponents in Level of Personality Functioning Scale	No formal (sub)components; examples (i.e., not an exhaustive list) found in Essential Features / Aspects of Personality Functioning
*Identity*	Identity
Sense of self; clarity of boundaries	Sense of self
Self-esteem; accuracy of self-appraisal	Self-worth; accuracy of self-view
Experience, tolerance, and regulation of emotion	Appropriate range/ awareness of emotions and their expression
*Self-direction*	Self-direction
Quality of goal directedness	Ability to plan, choose, and implement goals
Appropriateness of personal standards	Appropriateness of goals
Capacity to reflect productively on internal experience	Behavior modulation based on situation/ potential consequences
*Empathy*	–
Ability to appreciate and understand others’ experiences and motivations	Accuracy of interpersonal appraisal
Ability to understand and appreciate others’ perspectives	Ability to understand others’ perspectives
Awareness of effect of own actions on others	Appropriate behavior in response to emotions and stress
*Intimacy*	–
Ability to establish/ maintain satisfying, enduring relationships in personal and community life	Ability to develop and maintain close and mutually satisfying relationships
Desires and has caring, close, and reciprocal relationships	Interest in engaging in relationships with others
Strives for cooperative, mutually beneficial relationships	Ability to manage conflict in relationships
**Criterion B**	**Essential Feature**
One or more pathological personality traits Trait definition: A tendency to feel, perceive, behave, and think in relatively consistent ways across time, situations	The disturbance is manifest in patterns of cognition, emotional experience, emotional expression, and behavior that are maladaptive (e.g., inflexible or poorly regulated)
Pathological personality traits are organized into five broad domains:	To describe the most prominent characteristics of individuals’ personality that contribute to personality disturbance, optional domain specifiers are:
Negative Affectivity	Negative Affectivity
Detachment	Detachment
Antagonism	Dissociality
Disinhibition	Disinhibition
Psychoticism	–
–	Anankastia (obsessive–compulsive traits)
25 specific trait facets are also provided. Each trait domain is indexed by three specific facets; the other 10 facets can be used to characterize individuals’ personality further Descriptions of the trait domains and facets are in a table.	Traits domains do not formally have facets. However, there are facet-like descriptions of each trait domain in the Clinical Description and Diagnostic Requirements version of ICD-11 Descriptions of the trait domain specifiers are provided in text.
**Criterion C**	**Essential Feature**
Impairments in personality functioning and trait expression are relatively inflexible and pervasive across a broad range of personal and social situations.	The disturbance is manifest and across a range of personal and social situations. N.B. “inflexible or poorly regulated” are included under Essential Features associated with traits
**Criterion D**	**Essential Feature**
Impairments in personality functioning and trait expression are relatively stable across time, with onsets that can be traced back to at least adolescence or early adulthood	The disturbance has persisted over an extended period of time (e.g., lasting 2 years or more). ** Course Feature**: The disturbance tends to appear first in childhood, increase during adolescence, and continue into adulthood.
**Criterion E**	**Essential Feature**
Impairments in personality functioning and trait expression are not better explained by another mental disorder	The symptoms … are not better accounted for by another mental disorder.
**Criterion F**	**Essential Feature**
Impairments in personality functioning and trait expression are not solely attributable to the physiological effects of a substance or another medical condition.	The symptoms … are not due to the direct effects of a medication or substance, including withdrawal effects, and are not better accounted for by … a Disease of the Nervous System, or another medical condition.
**Criterion G:**	**Essential feature**:
Impairments in personality functioning and trait expression are not better understood as normal for an individual’s developmental stage or sociocultural environment.	Personality disorder should not be diagnosed if the patterns of behavior characterizing the personality disturbance are developmentally or can be explained primarily by social or cultural factors, including socio-political conflict.
**Severity of Impairment**	
The Level of Personality Functioning Scale uses the four Criterion A subcomponents and each of their three subcomponents to differentiate five impairment levels, Moderate or greater impairment is required to diagnose personality disorder,	Once a personality diagnosis has been established (based on having all the Essential [Required] Features), it should be described in of its level of severity. The aspects of personality functioning that contribute to determining the severity level, and examples for level are provided in the text. The 6D10.x codes below are separate personality disorder diagnoses.
1 = Some impairment (subthreshold)	QE50.7 Personality Difficulty (subthreshold)
2 = Moderate impairment	6D10.0 Mild Personality Disorder
3 = Severe impairment	6D10.1 Moderate Personality Disorder
4 = Extreme impairment	6D10.2 Severe Personality Disorder
**Specific PDs**	
Antisocial personality disorder	–
Avoidant personality disorder	–
Borderline personality disorder	An optional Borderline pattern specifier may be applied to individuals whose pattern of personality disturbance is characterized by a pervasive pattern of instability of interpersonal relationships, self- image, and affects, and marked impulsivity as indicated by many of the criteria for DSM-5, Section II Borderline personality disorder
Narcissistic personality disorder	–
Obsessive–compulsive personality disorder	–
Schizotypal personality disorder	Schizotypal Disorder is included in the chapter on Schizophrenia and Other Psychotic Disorders rather than in the personality disorder chapter
**Personality Disorder–Trait Specified**	
Personality Disorder–Trait Specified is the diagnosis to be given when the General Criteria for Personality Disorder—but not those for any specific personality disorder—are met.	Any personality disorder diagnosis for which one or more optional trait specifiers are applied is an ICD-11 “equivalent” of a *DSM-5* Personality Disorder-Trait Specified, although that is not an actual ICD-11 diagnosis.

*DSM-5* = Diagnostic and Statistical Manual of Mental Disorders, 5th Edition (American Psychiatric Association, 2013). *ICD-11* = International Classification of Diseases, 11th Edition (World Health Organization, 2022)

The differences summarized above and detailed in Table 1 are largely due to the different purposes and constituencies of the documents [7]. More specifically, (1) the *ICD-11* is designed to be used globally, in all countries that are member states of the World Health Organization. By international treaty, these 193 countries have agreed to use the ICD and its codes for reporting national health information. This agreement ensures that the reported data will be internationally comparable for communicating about diseases and health conditions (note that the ICD covers all medical diagnoses, not just mental disorders). Moreover, because WHO’s ultimate objective is “the attainment by all peoples of the highest possible level of health”[8], p. 2, a public-health focus is fundamental to the goals and internal organization of the ICD.

Given this focus, the *ICD-11* must be useful across the wide range of economic levels in countries around the globe, including in low-income countries, such as Afghanistan, Somalia, and Haiti (~ 15–20% of WHO member states), where general and community health-care workers, as well as traditional healers most commonly provide the only health care available; specialized mental-health professionals (e.g., psychiatrists, clinical psychologists) are very rare Thus, diagnostic requirements in the ICD across the spectrum of medical—including mental-health—disorders must be phrased so as to be understandable and applicable by health-care workers around the world.

That said, it is important to note that, unlike the DSM, there are multiple versions of the ICD. The simplest, most basic version is the coding tool, which simply lists all diagnoses and their codes (https://icd.who.int/ct/icd11_mms/en/release). If internet access is available, a key word or words can be entered online and all diagnoses with that key word or words will be provided along with their codes. For example, if one enters the key words “mental disorder,” the tool returns 50 entries that contain both words in either their title or their brief description, sorted by “Matching score” (i.e., likely relevance), beginning with “Mental, behavioral or neurodevelopmental disorder, unspecified. Mental disorder NOS” and ending with “Secondary psychotic syndrome. Psychotic syndrome due to health condition not classified under mental and behavioral disorders.” If internet access is unavailable, the WHO provides assistance.

A second version is the Clinical Description and Diagnostic Requirements (CDDR; formerly Guidelines, CDDG), which is the version referenced in this article and the one most commonly used globally in clinical settings, especially those in higher income countries. However, some countries, including the U.S., adapt the version for use by their constituents. In 2015, the U.S. switched to such a clinical modification based on *ICD-10* (*ICD-10-CM*), 15 years after it was ratified by WHO. It is unclear when the U.S. will shift to requiring the *ICD-11*.

Finally, a version with diagnostic criteria for research (DCR) is developed for mental and behavioral disorders. This version is still in progress for *ICD-11*, but presumably will be developed using similar methods to those used for *ICD-10-DCR* [9], which involved work in collaboration with world-leading experts and testing by researchers and clinicians in 32 countries who represented various traditions and schools of psychiatry. This version is the most similar to the *DSM*.

## Assessment of the *DSM-5, Section III *and *ICD-11* Personality Disorder Models

### AMPD

#### Personality Traits

The Personality Inventory for *DSM-5* [10], a self-report measure to assess the 25 personality trait facets—organized into five domains—of AMPD Criterion B, was published in conjunction with *DSM-5*. Since then, hundreds of articles using the measure have appeared including short forms (e.g., [11]); translations into multiple languages, including many European, Middle Eastern, Asian, and Slavic languages; two meta-analyses of 27 structural analyses (see [12]) and, more broadly, construct validity analyses in various sample types, including patients (e.g., [13]), adolescents (e.g., [14]), and older adults [15]; clinical utility analyses (e.g., [16]) and summary reviews of these literatures (e.g., [17, 18]).

The structural meta-analyses of the PID-5 facets (1) support APA’s (2013b) identification of three core facets for each of the five domains, (2) identified six additional facets that are moderately strong markers (loadings ≥ 0.40 and ≤ 0.60) of DET (restricted affectivity, depressivity), ANT (attention seeking, callousness, and hostility), and DIS (risk taking), and (3) identified three additional facets that are moderate markers (loadings ≥ 0.30 and ≤ 0.45) of NA (perseveration, rigid perfectionism, and submissiveness) and one facet (suspiciousness) that did not load > 0.35 on any domain (see [12] for a summary.)

Analyses of the PID-5 facets with measures of the five-factor model (FFM) of personality have demonstrated clear convergent-discriminant patterns for all but four facets: Submissiveness’ highest median loading was 0.38, whereas anhedonia, hostility, and distractibility each had a significant cross loading on NA. In addition, analyses with personality trait measures other than those of the FFM identified 13 traits with maladaptive content that is not well assessed by the PID-5 (e.g., measures of health anxiety and dependency would augment NA; greediness, domineeringness, and rudeness would enhance ANT; norm violation would expand DIS, and fantasy proneness would amplify PSY). Moreover, with the addition of workaholism, hypercontrol, and inflexibility, a sixth domain of Anankastia might emerge along with restricted affectivity (a secondary DET marker) and rigid perfectionism (a moderate NA marker). See review [12] for details. It is worth noting that there is also a structured interview for AMPD traits [19], but few studies have yet reported on its properties.

#### Personality Functioning

The assessment literature on AMPD Criterion A has accrued more slowly for two main reasons: (1) no self-report measure of Criterion A was published in conjunction with *DSM-5* and (2) unlike personality traits, personality functioning as a construct did not have an extensive, well-established research literature on which to build. In 2016, the first semi-structured interview [20] and self-report measure [21] of Criterion A were published. Since then, two other semi-structured interviews and at least five other self-report measures have been developed, including one for adolescents and one for Spanish-speaking Latin Americans (see review [22]).

Joint analyses of multiple self-report Criterion A measures have found a clear general factor [18] and have supported the measures’ convergent validity [23], but also have found differences in content and severity [24] and raised concerns regarding discriminant validity both among Criterion A measures [24] and with measures of Criterion B [22] (as well as among Criterion B measures). Morey and colleagues [25] demonstrated that the discriminant validity among Criterion B measures could be improved by controlling the variance of core dysfunction (i.e., Criterion A) that was common across traits.

An important theoretic-empirical question that remains undecided is the degree of overlap that “should” exist both among and between measures of Criterion A and B. That is, given that maladaptive traits are the overt expressions of personality dysfunction, some degree of overlap (i.e., cross-correlation) is expected, but a high degree of overlap would indicate redundancy and poor discriminant validity. However, there is not yet consensus on the “theoretically ideal” degree of overlap.

The multimethod literature on the AMPD is sparce. In a comprehensive literature review, [18] reported that of 36 studies that assessed Criterion A, self-report or another method (e.g., interview, informant report) were used roughly equally (41.6% vs. 44.4%), whereas 14% used more than one method. Of 229 studies that assessed Criterion B, 93% used only self-report, 5.0% used another method, and 6.4% used more than one method. Taken together, only 5.9% of studies assessing one or both criteria used more than one method. There appears to be reasonably good convergence across methods, especially between self-report and semi-structured interviews which, of course, both rely on the interviewee as the source of data. Moreover, self-informant agreement appears to be in the same moderate range as that found for normal-range traits and to vary systematically based on such factors as trait observability and the self-informant relationship (e.g., [26]), but little more can be said at this point. Clearly, more multi-method studies are needed to determine the best ways to evaluate personality traits and functioning for different purposes, including treatment planning, behavioral prediction, and prognosis.

### ICD-11 Personality Disorder Model

#### Personality Functioning

Despite *ICD-11*’s comparatively recent release, a number of measures of *ICD-11* PD have been developed (see overview [27]). However, only three of these measures assess the primary diagnostic requirement for *ICD-11* PD—problems in personality functioning; the others all assess the optional trait specifiers. One of the functioning measures—the Standardized Assessment of Severity of Personality Disorder (SASPD; [28]) is a modification of a brief screening measure developed to assess PD severity based on the *DSM-IV/ ICD-10* categorical PD model [29]. However, it was based on an early draft of the final *ICD-11* PD model, and studies have raised concerns about its validity (e.g., see review [30]). Another *ICD-11* personality functioning measure is preliminary and still under development [27].

The last measure is based on the CDDR for *ICD-11* PD [31]. Ten of its items (four each assessing self-functioning and interpersonal functioning, and three items assessing, respectively, emotional, cognitive, and behavioral difficulties) have five response options that define a bipolar scale, with the mid-point reflecting healthy functioning. For example, the extremes of the item assessing behavioral control are “I often act so rashly and impulsively that it causes serious problems” and “I am often so overcontrolled in my actions that I hardly get anything out of life,” with the middle option being “I am generally able to be spontaneous while keeping appropriate control of my emotions.” Bach and colleagues [31] reported that the measure was largely unidimensional, had moderately strong correlations with other self-report measures of personality functioning and traditional categorical PD scores (median *r* = 0.57), and a moderately strong correlation (*r* = 0.60) with clinician-rated *ICD-11* PD diagnosis. Brown and Sellbom [30] replicated this finding has been by (*r* = 0.61) and also reported a correlation of 0.63 with the overall score of a structured interview for personality functioning.

#### Optional Personality Trait Specifiers

In contrast to the scarcity of measures of the only essential feature of *ICD-11* PD personality functioning), there are at least 10 measures that assess the optional trait specifiers, seven of which are adaptations of pre-existing measures, including five based on the PID-5 (see review [27]). Oltmanns and Widiger developed the three measures that are not adaptations: the 60-item Personality Inventory for ICD-11 (PICD-11; [32]), based on a preliminary description of the trait specifiers, an informant version of this measure (IPiCD-11[33]), and the 121-item Five-Factor Personality Inventory for *ICD-11* (FFiCD [34]), which is the only measure of which I am aware that also assesses the borderline pattern specifier. The non-PID-5 adaptations were based on the Personality Assessment Schedule (PAS [35]) and include the 40-item PAS *ICD-11* and a 17-item Korean version, the Personality Assessment Questionnaire for *ICD-11* (PAQ-11; [36]). The five PID-5 adaptations range from 34 to 158 items, with the most noteworthy being perhaps the 36-item PID-5-BF+ [37]. It is a modification of the 34-item PID5-BF+ [38], assesses the combined six domains of the AMPD and *ICD-11*, was developed by an international group, and has 12 versions in various European languages and Brazilian Portuguese.

To date, the only measure of which I am aware that assesses both *ICD-11* PD’s required personality functioning criteria and its optional trait specifiers is Clark and colleagues’ preliminary measure [27], which is in the revision process. When finalized, there are plans to translate it into multiple languages including Afrikaans, Danish, German, Italian, Korean, Persian, Portuguese, Serbian, Sesotho, Russian, Spanish, Swedish and, it is to be hoped, other languages as well.

## Whither PD? Future Developments of the AMPD and ICD-11 Personality Disorder Model

Given that ICD-11 has already shifted to a dimensional PD model, and the CDDR have been published, the only remaining step is development of the Research version. At present, there is no public information regarding when that will occur and, in fact, may already have begun. Therefore, the potential for change in the *DSM-5* from a categorical to dimensional model is the “$64,000 question.”^1^

Many who had been hoping that the *DSM-5* would finally include the first dimensional PD model were disappointed, to say the least, when the *DSM-IV* PD model was carried over completely intact into *DSM-5*. Nonetheless, that an alternative dimensional model was also included in the manual represented a major step forward—one that, in hindsight, was likely a better outcome than putting the AMPD in the main “Section II.” I say this because although the AMPD was firmly based in existing research on personality functioning and traits, and had positive results in the field trials, its particular instantiation had not been fully vetted either by research or clinically. As such, had the AMPD replaced the existing categorical model in *DSM-5*, there likely would have been an uproar in the field concerning the difficulty of implementing what to many, perhaps most, psychiatrists, as well as many clinical psychologists and other mental health professionals, was an unfamiliar system.

Now, more than 10 years later, the field is in a very different position. A plethora of measures have been developed and used in hundreds of research studies on multiple aspects of the AMPD, which generally have shown the measures and the model to have strong construct validity (e.g., see a comprehensive review [18], and the 10-year retrospective issue of *Personality Disorders: Theory, Research, and Treatment* (PDTRT) [39]. Moreover, the concurrent and predictive validity of the AMPD have proved equivalent or stronger than the current system (see the 2024 Special Issue of PDTRT [40]). In addition, the newly formed Hierarchical Taxonomy of Psychopathology (HiTOP) consortium [41], has proffered a comprehensive dimensional diagnostic system for mental disorder that has rapidly developed a substantial research literature, including 16 articles in *World Psychiatry*, 15 in *Clinical Psychological Science*, and 11 in *Journal of Psychopathology and Clinical Science* (formerly *Abnormal Psychology*), so the AMPD’s dimensional approach to diagnosis can no longer be considered an anomaly.

In late fall, 2021, the APA began a process that is still ongoing to consider whether a dimensional PD diagnostic system should replace the current categorical system in *DSM-5* Section II. To this end, the *DSM-5* Revision Steering Committee (SC) convened a panel of ~ 20 PD research and clinical experts, including both psychiatrists and psychologists. A series of meetings were held and by late summer, 2022, a consensus emerged that such an action might be warranted. The panel was then divided into four subgroups, which were charged with conducting literature reviews on, respectively, Criterion A personality functioning, Criteria B traits, the AMPD as a whole, and the current categorical model. Before this work could begin, however, subgroup members required vetting by APA Governance for conflicts of interest, which took until March, 2023. The subgroup literature reviews were then completed by early fall, 2023 and circulated to the original larger panel and *DSM-5* SC for discussion.

The discussions were cordial although there clearly remained differences of opinion. Nonetheless, it ultimately was decided that the SC Chair would appoint a subset of panel members to draft a proposal for implementing a dimensional PD diagnostic model in Section II following the guidelines required of all proposals to amend the DSM-5.^2^ In February, 2024, the group submitted the first draft of their proposal to the *DSM-5* SC. Over the next few months, an iterative back-and-forth process ensued between the proposal group and the SC, at the end of which the SC voted to send the proposal to the Review Committee (RC) whose charge includes personality disorder. Importantly, soon after doing so, it was time for many members of the SC, including the Chair, to rotate off, and new members have been appointed, so a somewhat different SC will carry the process forward. As of this writing, the RC is proceeding through its process, which includes getting input from a range of PD research experts and clinicians, including individuals who are likely to favor—and others to oppose—the proposal. There is no specified timeline for proposal reviews, so when the RC will complete its work is indeterminate.

If the RC recommends to the SC that the proposal move forward—with or without modification—the SC will post the proposal for ~ 45 days of public comment. Before doing so, the SC may discuss the recommended modifications with the proposers, and the proposal may be revised through another iterative process. If the RC recommends against the proposal, the SC can move forward anyway, but that is extremely unlikely. After the public comment period, the proposal and comments will be sent back to the RC to decide whether it wants to revise its recommendations, after which it goes back to the SC for final review, with again the possibility of an iterative process with the proposers, which could even include requests for additional data or new analyses. Assuming the SC ultimately recommends approval, the (likely revised) proposal and public comments are forwarded to the APA Assembly for review and, if they agree, to the APA Board of Trustees, which has the final say. Thus, in conclusion, all that can be said as of this writing is that major change in the *DSM-5* PD diagnostic system is being considered and is possible in the relatively near future, but the process is necessarily quite thorough and deliberate, not to mention time-consuming, and the outcome is by no means guaranteed.

## Conclusion

The PD world is continuing to evolve and in the past 10 years has changed more than it did in the previous quarter century. In 2013, the American Psychiatric Association decided to take the interim step of introducing a dimensional PD model as an alternative model alongside the categorical one, which allowed (1) the new model to develop a research base and (2) exploration of the new model’s clinical utility. Building on the growing evidence supporting the AMPD, the World Health Organization decided to skip the interim step and jump straight to introducing a dimensional PD model in *ICD-11*, one that is similar to the AMPD, but better suited to the global population that is its focus. The next 10 years will likely see a dimensional PD formally instantiated in *DSM-5* as well as *ICD-11*, although both, no doubt, will continue to evolve as their research bases expand and as clinical experience with the models becomes widespread.

## Key References

- Clark LA, Cuthbert B, Lewis-Fernández R, Narrow WE, Reed GM. Three approaches to understanding and classifying mental disorder: ICD-11, DSM-5, and the National Institute of Mental Health’s Research Domain Criteria (RDoC). Psychological Science in the Public Interest 2017 09;18(2):72–145. 10.1177/1529100617727266.
  - Provides histories and descriptions of ICD and DSM diagnostic classification systems, and the NIMH’s Research Domain Criteria (RDoC). The two nosologies are relevant to clinical diagnosis and service provision, as well as public health and research, whereas RDoC is a framework to integrate basic behavioral and neuroscience research to further understanding of psychopathology. Discusses four key challenges to understanding and classifying mental disorder: etiology, categories or dimensions, thresholds, and comorbidity.
- Brown, T. A., & Sellbom, M. (2023). Further validation of the Personality Disorder Severity for ICD-11 (PDS-ICD-11) scale in a community mental health sample. *Psychological Assessment, 35*(8), 706–714. 10.1037/pas0001253.
  - Reports validity and clinical utility data for the PDS-ICD-11 in an outpatient sample. The measure is arguably the currently best self-report measure available of the ICD-11 PD model’s primary requirement of personality functioning impairment in three main levels of severity: mild, moderate, and severe. Findings include moderate-to-large correlations with clinician ratings, across all severity levels, but more variable associations with self-report and informant-report measures.
- Bach B, Kerber A, Aluja A, Bastiaens T, Keeley JW, Claes L, et al. International assessment of *DSM-5* and *ICD-11* personality disorder traits: Toward a common nosology in DSM-5.1. Psychopathology 2020;53(3–4):179–188. 10.1159/000507589.
  - Reports on an international group’s development and initial validation of a 36-item measure to assess the combined six domains of AMPD and ICD-11 traits in 12 languages. Findings include strong and consistent structures across three of the translations, good discriminant validity among the scales, with all *r*s < 0.40, and strong internal consistency reliability across translations. The measure provides a way to assess both dimensional PD models’ traits, including three facets per domain and thus can facilitate international PD research.
- Bach B, Tracy M. Clinical utility of the alternative model of personality disorders: A 10th year anniversary review. Personality Disorders: Theory, Research, and Treatment 2022 07;13(4):369–379. 10.1037/per0000527.
  - A narrative review of the AMPD clinical-utility literature, including clinician and patient acceptability, ease of use, diagnostic accuracy, continuity with familiar clinical constructs, and usefulness in treatment planning, case-formulation, and clinical management. The authors identify area for improvement, including eliminating the hybrid PD categories and the need for studies of the model’s value for clinical resource allocation, clinician-patient and family communication, and treatment selection.
- Clark LA, Watson D. The trait model of the DSM–5 alternative model of personality disorder (AMPD): A structural review. Personality Disorders: Theory, Research, and Treatment 2022 07;13(4):328–336. 10.1037/per0000568.
  - Reviews the structural literature on the PID-5, both its internal structure and relations with the five-factor model of personality. The literature confirms that the best three facets for each domain are those recommended by the measure’s authors and APA, identifies secondary domain markers, interstitial facets and facets that do not load strongly on any factor, as well as important constructs that the AMPD does not—but should—include. Ends with 11 recommendations for improving the AMPD trait model.
- Morey LC, McCredie MN, Bender DS, Skodol AE. Criterion A: Level of personality functioning in the alternative DSM–5 model for personality disorders. Personality Disorders: Theory, Research, and Treatment 2022 07;13(4):305–315. 10.1037/per0000551.
  - Reviews the literature on AMPD Criterion A, including a brief history of its conceptualization and development and the most important advances in its measurement, with a focus on the empirical literature on the Level of Personality Functioning Scale (LPFS) primarily in interview format but with some discussion of self-report measures. Results have generally demonstrated that the LPFS can be rated reliably by both experienced and inexperienced raters, has strong associations with relevant criterion variables and has both predictive validity and clinical utility.
- Morey LC, Good EW, Hopwood CJ. Global personality dysfunction and the relationship of pathological and normal trait domains in the DSM-5 alternative model for personality disorders. J Pers 2022 02;90(1):34–46. 10.1111/jopy.12560.
  - Examines interrelations among measures of AMPD personality functioning impairment, and personality traits in the maladaptive and typical ranges. Results suggest that current measures of maladaptive-range trait expression reflect the variance in measures of both core personality dysfunction and typical-range traits. This is bolstered by findings that problems in discriminant validity between maladaptive- and typical-range traits largely disappear when their common personality dysfunction variance is controlled.
- Waugh MH, McClain CM, Mariotti EC, Mulay AL, DeVore EN, Lenger KA, et al. Comparative content analysis of self-report scales for level of personality functioning. J Pers Assess 2021 Mar;103(2):161–173. 10.1080/00223891.2019.1705464.
  - Obtained ratings of eight measures of AMPD personality functioning (Criterion A) including four that were specifically developed to target AMPD Criterion A (one with subscales for self and interpersonal functioning; three with subscales for Identity, Self-direction, Empathy, and Intimacy), and four that had been developed prior to its publication. Results provide important new information for future research and for measure selection in clinical practice.
- Zimmermann J, Kerber A, Rek K, Hopwood CJ, Krueger RF. A brief but comprehensive review of research on the alternative DSM-5 model for personality disorders. Current Psychiatry Reports. 2019 Sep;21:1–9. 10.1007/s11920-019-1079-z.
  - This review of 237 publications on AMPD Criteria A and B reports that measures of these criteria have acceptable interrater reliability and consistent structures, converge meaningfully with various external measures, and show evidence of added predictive power over categorical PD diagnoses. Nonetheless, measures of criterion A correlate strongly with those of Criterion B, raising theoretico-empirical challenges.

## Data Availability

No datasets were generated or analysed during the current study.

## Footnotes

1 An idiom for “the most important question,” based on a 1950s American game show in which the final question was worth $64,000.

2 https://www.psychiatry.org/psychiatrists/practice/dsm/submit-proposals

## References

[1] American Psychiatric Association. Diagnostic and Statistical Manual of Mental Disorders. 5th ed. Arlington, VA: Author; 2013.

[2] World Health Organization (WHO). International classification of diseases, Tenth Revision (ICD-10). 1993. https://www.who.int/publications/i/item/9241544554

[3] American Psychiatric Association. *Personality Inventory for DSM-5, Full Version-Adult*. https://www.psychiatry.org/FileLibrary/Psychiatrists/Practice/DSM/APA_DSM5_The-Personality-Inventory-For-DSM-5-Full-Version-Adult.pdf

[4] Clark LA, Vanderbleek E, Shapiro J, Nuzum H, Allen X, Daly E, et al. The brave new world of personality disorder-trait specified: Effects of additional definitions on prevalence and comorbidity. Psychopathol Rev. 2015;2(1):52–82. 10.5127/pr.036314. 26097740 10.5127/pr.036314 PMC4469240

[5] Clark LA, Ro E, Nuzum H, Vanderbleek EN, Allen X. Personality disorder coverage, prevalence, and convergence: Do the DSM-5’s two models of personality disorder identify the same patients? Psychol Med. 2024;54:2010–21. 10.1017/S0033291724000357. 10.1017/S0033291724000357 PMC11413344 38501282

[6] World Health Organization (WHO). International classification of diseases, Eleventh Revision (ICD-11). 2019/2022. https://icd.who.int/. License: CC BY-ND 3.0 IGO

[7] Clark LA, Cuthbert B, Lewis-Fernández R, Narrow WE, Reed GM. Three approaches to understanding and classifying mental disorder: ICD-11, DSM-5, and the National Institute of Mental Health’s Research Domain Criteria (RDoC). Psychol Sci Public Interest. 2017;18(2):72–145. 10.1177/1529100617727266. 29211974 10.1177/1529100617727266

[8] World Health Organization. Constitution of the World Health Organization, in World Health Organization Basic documents. 49th ed. Geneva: World Health Organization; 2020. p. 2–19. https://apps.who.int/gb/bd/pdf_files/Bd_49th-en.pdf.

[9] World Health Organization. The ICD-10 classification of mental and behavioural disorders: diagnostic criteria for research. World Health Organization; 1993. https://www.who.int/publications/i/item/9241544554

[10] Krueger RF, Derringer J, Markon KE, Watson D, Skodol AE. Initial construction of a maladaptive personality trait model and inventory for DSM-5. Psychol Med. 2012;42(9):1879–90. 10.1017/S0033291711002674. 22153017 10.1017/S0033291711002674 PMC3413381

[11] Maples JL, Carter NT, Few LR, Crego C, Gore WL, Samuel DB, et al. Testing whether the DSM-5 personality disorder trait model can be measured with a reduced set of items: an item response theory investigation of the Personality Inventory for DSM-5. Psychol Assess. 2015;27(4):1195–1210. 10.1037/pas0000120. 10.1037/pas0000120 25844534

[12] Clark LA, Watson D. The trait model of the DSM–5 alternative model of personality disorder (AMPD): a structural review. Personality Disorders: Theory, Res, Treatment. 2022;13(4):328–36. 10.1037/per0000568. 10.1037/per0000568 35787115

[13] Bastiaens T, Claes L, Smits D, De Clercq B, De Fruyt F, Rossi G, et al. The construct validity of the Dutch Personality Inventory for DSM-5 Personality Disorders (PID-5) in a clinical sample. Assessment. 2016;23(1):42–51. 10.1177/1073191115575069. 25736039 10.1177/1073191115575069

[14] De Clercq B, De Fruyt F, De Bolle M, Van Hiel A, Markon KE, Krueger RF. The hierarchical structure and construct validity of the PID-5 trait measure in adolescence. J Pers. 2014;82(2):158–69. 10.1111/jopy.12042. 23647646 10.1111/jopy.12042

[15] Debast I, Rossi G, van Alphen SPJ. Construct validity of the DSM-5 section III maladaptive trait domains in older adults. J Personal Disord. 2017;31(5):671–88. 10.1521/pedi_2017_31_274. 10.1521/pedi_2017_31_274 28072042

[16] Krueger RF, Hobbs KA. An overview of the DSM-5 alternative model of personality disorders. Psychopathology. 2020;53(3–4):126–32. 10.1159/000508538. 10.1159/000508538 PMC7529724 32645701

[17] Bach B, Tracy M. Clinical utility of the alternative model of personality disorders: a 10th year anniversary review. Personality Disorders: Theory, Res, Treatment. 2022;13(4):369–79. 10.1037/per0000527. 10.1037/per0000527 35787123

[18] Zimmermann J, Kerber A, Rek K, Hopwood CJ, Krueger RF. A brief but comprehensive review of research on the alternative DSM-5 model for personality disorders. Curr Psychiatry Rep. 2019;21:1–9. 10.1007/s11920-019-1079-z. 10.1007/s11920-019-1079-z 31410586

[19] First MB, Skodol AE, Bender DS, Oldham JM. Module II: structured clinical interview for personality traits. In: First MB, Skodol AE, Bender DS, Oldham JM, editors. Structured clinical interview for the DSM-5® alternative model for personality disorders (SCID-5-AMPD). American Psychiatric Association. 2018.

[20] Thylstrup B, Simonsen S, Nemery C, Simonsen E, Noll JF, Myatt MW, et al. Assessment of personality-related levels of functioning: a pilot study of clinical assessment of the DSM-5 level of personality functioning based on a semi-structured interview. BMC Psychiatry. 2016;12(16):1-8. 10.1186/s12888-016-1011-6. 10.1186/s12888-016-1011-6 PMC5000451 27562651

[21] Hutsebaut J, Feenstra DJ, Kamphuis JH. Development and preliminary psychometric evaluation of a brief self-report questionnaire for the assessment of the DSM–5 level of personality functioning scale: the LPFS Brief Form (LPFS-BF). Personality Disorders: Theory, Res, Treatment. 2016;7(2):192–7. 10.1037/per0000159. 10.1037/per0000159 26595344

[22] Morey LC, McCredie MN, Bender DS, Skodol AE. Criterion A: level of personality functioning in the alternative DSM–5 model for personality disorders. Personality Disorders: Theory, Res, Treatment. 2022;13(4):305–15. 10.1037/per0000551. 10.1037/per0000551 35787111

[23] McCabe GA, Oltmanns JR, Widiger TA. Criterion A scales: convergent, discriminant, and structural relationships. Assessment. 2021;28(3):813–28. 10.1177/1073191120947160. 10.1177/1073191120947160 32749149

[24] Waugh MH, McClain CM, Mariotti EC, Mulay AL, DeVore EN, Lenger KA, et al. Comparative content analysis of self-report scales for level of personality functioning. J Pers Assess. 2021;103(2):161–73. 10.1080/00223891.2019.1705464. 10.1080/00223891.2019.1705464 31917602

[25] Morey LC, Good EW, Hopwood CJ. Global personality dysfunction and the relationship of pathological and normal trait domains in the DSM-5 alternative model for personality disorders. J Pers. 2022;90(1):34–46. 10.1111/jopy.12560. 32422689 10.1111/jopy.12560

[26] Yalch MM, Hopwood CJ. Target-, informant-, and meta-perceptual ratings of maladaptive traits. Psychol Assess. 2017;29(9):1142–56. 10.1037/pas0000417. 10.1037/pas0000417 27831728

[27] Clark LA, Corona-Espinosa A, Khoo S, Kotelnikova Y, Levin-Aspenson H, Serapio-García G, et al. Preliminary scales for ICD-11 personality disorder: Self and interpersonal dysfunction plus five personality disorder trait domains. Front Psychol. 2021;12(12):16. 10.3389/fpsyg.2021.668724. 10.3389/fpsyg.2021.668724 PMC8311289 34322060

[28] Olajide K, Munjiza J, Moran P, O’Connell L, Newton-Howes G, Bassett P, et al. Development and psychometric properties of the Standardized Assessment of Severity of Personality Disorder (SASPD). J Personal Disord. 2018;32(1):44–56. 10.1521/pedi_2017_31_285. 10.1521/pedi_2017_31_285 28513349

[29] Moran P, Leese M, Lee T, Walters P, Thornicroft G, Mann A. Standardised assessment of personality - abbreviated scale (SAPAS): preliminary validation of a brief screen for personality disorder. British J Psychiatry. 2003;183(3):228–32. 10.1192/bjp.183.3.228. 10.1192/bjp.183.3.228 12948996

[30] Brown TA, Sellbom M. Further validation of the personality disorder severity for ICD-11 (PDS-ICD-11) scale in a community mental health sample. Psychol Assess. 2023;35(8):706–14. 10.1037/pas0001253. 10.1037/pas0001253 37384513

[31] Bach B, Brown TA, Mulder RT, Newton-Howes G, Simonsen E, Sellbom M. Development and initial evaluation of the ICD-11 Personality Disorder Severity Scale: PDS-ICD-11. Personality Mental Health. 2021;15(3):223–36. 10.1002/pmh.1510. 34002530 10.1002/pmh.1510

[32] Oltmanns JR, Widiger TA. A self-report measure for the ICD-11 dimensional trait model proposal: the personality inventory for ICD-11. Psychol Assess. 2018;30(2):154–69. 10.1037/pas0000459. 10.1037/pas0000459 PMC5930359 28230410

[33] Oltmanns JR, Widiger TA. The self- and informant-personality inventories for ICD-11: agreement, structure, and relations with health, social, and satisfaction variables in older adults. Psychol Assess. 2021;33(4):300–10. 10.1037/pas0000982. 10.1037/pas0000982 PMC8483599 33779193

[34] Oltmanns JR, Widiger TA. The five-factor personality inventory for ICD-11: a facet-level assessment of the ICD-11 trait model. Psychol Assess. 2020;32(1):60–71. 10.1037/pas0000763. 10.1037/pas0000763 PMC6928398 31414852

[35] Tyrer P, Alexander MS, Cicchetti D, Cohen MS, Remington M. Reliability of a schedule for rating personality disorders. Br J Psychiatry. 1979;08(135):168–74. 10.1192/bjp.135.2.168. 10.1192/bjp.135.2.168 497620

[36] Kim Y, Tyrer P, Hwang S. Personality assessment questionnaire for ICD-11 personality trait domains: Development and testing. Personality Mental Health. 2021;15(1):58–71. 10.1002/pmh.1493. 32638542 10.1002/pmh.1493

[37] Bach B, Kerber A, Aluja A, Bastiaens T, Keeley JW, Claes L, et al. International assessment of DSM-5 and ICD-11 personality disorder traits: Toward a common nosology in DSM-5.1. Psychopathology 2020;53(3–4):179–188. 10.1159/000507589 10.1159/000507589 32369820

[38] Kerber A, Schultze M, Müller S, Rühling RM, Wright AGC, Spitzer C, et al. Development of a short and ICD-11 compatible measure for DSM-5 maladaptive personality traits using ant colony optimization algorithms. Assessment. 2022;29(3):467–87. 10.1177/1073191120971848. 10.1177/1073191120971848 PMC8866743 33371717

[39] Sharp C, Miller JD. Ten-year retrospective on the DSM–5 alternative model of personality disorder: seeing the forest for the trees. Personality Disorders: Theory, Res, Treatment. 2022;13(4):301–4. 10.1037/per0000595. 10.1037/per0000595 35787110

[40] Sharp C, Miller JD. Head-to-head comparisons of diagnostic and statistical manual of mental disorders, 5th ed. Ssection II and Section III personality disorder in predicting clinical outcomes. Personality Disorders: Theory, Res, Treatment 2024;15(5):275. 10.1037/per0000691. 10.1037/per0000691 39235913

[41] Kotov R, Krueger RF, Watson D, Achenbach TM, Althoff RR, Bagby RM, et al. The hierarchical taxonomy of psychopathology (HiTOP): a dimensional alternative to traditional nosologies. J Abnorm Psychol. 2017;126(4):454–77. 10.1037/abn0000258. 10.1037/abn0000258 28333488

